# The mystery of the blue toe

**DOI:** 10.1515/rir-2025-0029

**Published:** 2025-12-27

**Authors:** Jing Wang, Dachen Zuo, Shengnan Yu, Sha Ma

**Affiliations:** Department of Rheumatology, The First People’s Hospital of Yunnan Province, The Affiliated Hospital of Kunming University of Science and Technology, Kunming, Yunnan Province, China

Dear Editor,

Cholesterol embolization syndrome (CES) is a rare clinical situation that could mimic the clinical presentation of systemic vasculitis. However, the differential between CES and systemic vasculitis is very important as the treatment strategy and prognosis is significantly different between these two conditions, Herein, we reported a case of CES who died from CES to alert rheumatologists to this rare syndrome.

**Figure 1:**
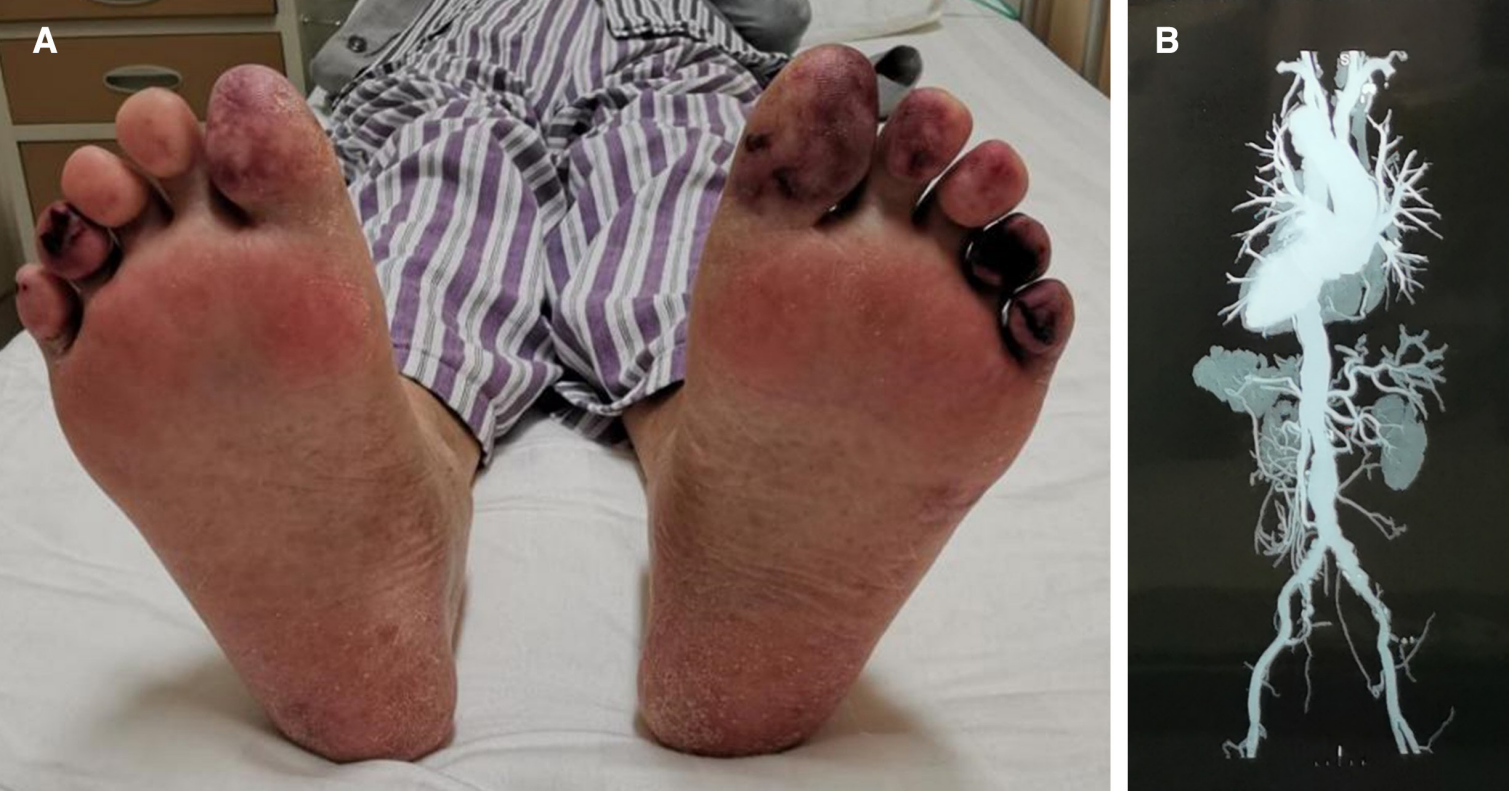
(A) blue toe. (B) The B shows calcified atherosclerotic plaque throughout the visualized thoracoabdominal aorta (arrow) on a three-dimensional computed tomography reconstruction.

A 73-year-old male was admitted to the rheumatology department due to blue discoloration and pain of his toes for half a month. The patient had a 50-year smoking history (20 cigarettes/day) and a history of atherosclerosis. Two months prior to admission, he underwent percutaneous coronary intervention (PCI). After the procedure, he experienced fatigue, anemia, and weight loss. Upon admission, the blood pressure was 165/86 mmHg and the bilateral dorsalis pedis artery were palpable. Blue toes were found during physical examination (Figure 1A). Laboratory tests revealed serum creatinine: 261 μmol/L, and elevated erythrocyte sedimentation rate (ESR: 96 mm/h) and C-reactive protein (CRP: 47.11 mg/L). His ANA (antinuclear antibodies), ANCA (antineutrophil cytoplasmic antibodies) and aPL (antiphospholipid anti-body) tests were negative. As the patient presented with renal insufficiency, along with a history of atherosclerosis (Figure 1B) and recent vascular intervention, the diagnosis of CES was highly suspected. Aspirin (100 mg/day), clopidogrel (75 mg/day), and aorvastatin (20 mg/day), prednisolone (40 mg/day) were prescibed. After 14 days of treatment, the patient’s toe pain was slightly relieved, his ESR decreased to 18 mm/h, but the serum creatinine level did not improve significantly. The patient refused further treatment and deceased two months later.

CES is a systemic disease caused by showering of atherosclerotic plaque materials, such as cholesterol crystals (CCs), from the aorta and its major branches to distal branches, leading to ischemic and inflammatory damage to multiple organs.^[1]^ CES is induced mostly by interventional and surgical procedures; however, it may also occur spontaneously.

It has been reported that the time from vascular interventional procedure to the appearance of clinical manifestations ranges from a few days to four months.^[2]^ Kidney is a frequent target organ for cholesterol emboli because its proximity to abdominal aorta and it receives an enormous amount of blood flows.^[3]^ Renal replacement therapy is required in 37–61% of CES patients.^[4]^

Although blue toe syndrome is a hallmark of CES, it can also occur in patients with vasculitis or endocarditis. CES may be considered as one of the “great imitators”. The manifestations of CES can resemble vasculitis with involvement of the skin (livedo-reticularis, purpura, blue toe), kidneys (acute kidney injury, uncontrolled hypertension), gastrointestinal tract (mesenteric ischemia), central nervous system (confusion, stroke) and eyes (involvement of retinal vessels, *i.e*., Hollenhorst plaques).^[1]^ If a patient has a delayed-onset of acute kidney injury (AKI) together with cutaneous manifestations, such as livedo reticularis or blue-toe syndrome, a clinical diagnosis of CES can be established.^[5]^

It is very important to make a differential diagnosis between CES and systemic vasculitis because their treatments and prognoses are different. CES predominantly affects older adults with advanced atherosclerosis, hypertension, or diabetes, often following vascular interventions. CES causes acute or subacute renal failure due to crystal-induced ischemic glomerulopathy, often with eosinophiluria and modest proteinuria. Vasculitis typically induces rapidly progressive glomerulonephritis with active urinary sediment (dysmorphic red blood cells [RBCs], red cell casts) and significant protein-uria. Systemic vasculitis is associated with ANCA positivity (in small-vessel vasculitis), or anti-glomerular basement membrane [anti-GBM] antibodies. CES lacks autoantibodies but may show eosinophilia, thrombocytopenia, or hypocomplementemia. Definitive diagnosis of CES requires demonstration of cholesterol clefts in arterioles (*e.g*., skin, kidney, or muscle biopsy), often with minimal inflammation. Vasculitis shows leukocytoclastic or granulomatous vascular inflammation on biopsy. Atherosclerotic plaque burden on angiography or vascular imaging supports CES, while vasculitis may reveal aneurysms, stenoses, or vessel wall edema on magnetic resonance imaging and computed tomography (MRI/CT).^[6]^

The mortality rate associated with CES is high (64% to 81%), particularly patients who develop renal failure or multi-organ involvement.^[7]^ Although it is difficult to completely eliminate the occurrence of cholesterol embolization syndrome, through the comprehensive management of risk factors (hypertension, hyperlipidemia, diabetes), careful medical procedures, and the use of anticoagulants and thrombolytics, can effectively reduce the incidence of CES.^[8]^ Statins can reduce the volume of atherosclerotic plaque and stabilize the plaque, so. ameliorate the renal and patient’s prognosis. ^[9]^ Anticoagulant therapy in CES is controversial and is generally not recommended. Anticoagulation does not improve arterial patency in the presence of CC debris or inflammatory arterial occlusion.^[10]^ In the short term, glucocorticoid treatment for CES patients improved their renal outcomes, but in the long term, the treatment was ineffective.^[11]^ Experimental studies indicated that colchicine could reduce cardiovascular events in high-risk patients and affects CC formation and breakdown. Interleukin [IL]-6 and IL-1β inhibitors also show promise results.^[12]^

In conclusion, due to CES’s frequent clinical manifestations including blue toe, livedo reticularis, and acute renal insufficiency along with elevated acute phase inflammatory markers such as ESR and CRP observed in some patients, this condition often mimics primary systemic vasculitis, leading to frequent patient admissions to rheumatology departments. Rheumatologists should consider CSE in the differential diagnosis of systemic vasculitis, especially in patients with severe atherosclerosis and those who have recently undergone endovascular intervention, vascular surgery, anticoagulation or thrombolysis.
